# Consequences of twinning induction to *Noemi* ewes by a recombinant human follicle-stimulating hormone compared with pituitary-derived porcine follicle-stimulating hormone on follicular dynamics, maternal biochemical attributes, and neonatal traits

**DOI:** 10.14202/vetworld.2020.633-641

**Published:** 2020-04-08

**Authors:** Moustafa Mohamed Zeitoun, Abdulrahman O. El-Dawas, Mohamed A. Ateah, Mohamed Ahmed Shehab El-Deen

**Affiliations:** 1 Department of Animal Production and Breeding, College of Agriculture and Veterinary Medicine, Qassim University 51452, Saudi Arabia; 2 Department of Animal and Fish Production, Faculty of Agriculture, El-Shatby, Alexandria University, Alexandria 21545, Egypt; 3 Department of Animal Production, Faculty of Agriculture, Suez Canal University, Ismailia 41522, Egypt

**Keywords:** blood metabolites, human follicle-stimulating hormone, porcine follicle-stimulating hormone, sheep, twinning

## Abstract

**Aim:**

The aim of this study was to investigate the effectiveness of using recombinant human follicle-stimulating hormone (FSH) compared with pituitary-derived porcine FSH given as one dose or multiple doses on the neonatal traits, follicular dynamics, and maternal blood biochemical constituents in *Noemi* ewes.

**Materials and Methods:**

A 3×2 factorial arrangement was designed utilizing 60 adults *Noemi* ewes to test the effects of using two sources of FSH (*human* vs*. porcine*) in addition to control, either given as a single total dose or six descending doses to provoke twinning. Six treatments (T) were tested (n=10 ewes/T). C1 and C6 served as control ewes given saline as one dose and six doses, respectively; H1 and H6 ewes were given human FSH as one and six doses; and P1 and P6 ewes were given porcine FSH similar to the above treatments. Saline and/or FSH administration were administered at days 8, 9, and 10 of the 10-day controlled internal drug release (CIDR) implant. At CIDR removal, fertile rams were used for natural mating. Blood samples for the assessment of serum metabolites were collected.

**Results:**

Twinning increased in FSH-treated ewes than control. However, giving FSH of either source as a single dose resulted in a higher incidence of stillbirths. Pregnancy rates were 30, 40, 50, 60, 70, and 80% in C1, C6, P1, P6, H1, and H6, respectively. Respective percent of ewes delivering twins/multiple birth was 0, 0, 80, 66.7, 71.4, and 87.5%. FSH of human source was more efficient for folliculogenesis than porcine FSH. Administration of FSH increased blood cholesterol, decreased high-density lipoprotein; however, low-density lipoprotein levels were not different than control. Moreover, an interaction (p<0.05) exists between source and type of FSH administration on blood glucose. Six doses of FSH elevated blood protein. Blood albumin decreased by porcine-FSH but not affected by human-FSH. Blood globulins were not different due to source of FSH, whereas giving FSH as six doses increased globulins than in single-dose protocol. Contrariwise, an interaction was found between source and type of FSH administration on elevating the activity of alanine aminotransferase and reducing the activity of aspartate aminotransferase.

**Conclusion:**

Administration of human FSH at 180 IU in six descending doses resulted in the best neonatal outcomes and maternal health in *Noemi* ewes.

## Introduction

Twinning of single-bearing animals is a focus of the reproductive physiology researchers to overcome the lack of animal protein in developing countries. Reproductive traits differ greatly among sheep breeds. *Noemi* sheep is a gulf native breed derived from *Awassi* sheep with a mean of 1.2 offspring per delivery [1]. Furthermore, *Najdi* is a predominant sheep breed originated in the central region of the Kingdom of Saudi Arabia with a mean litter size of 1.30 [2]. It is well known earlier that prolificacy of ewes is a genetic phenomenon [3], and heritability of multiple births has been found to be quite low, ranging from 0% to 20% [4]. Most of indigenous sheep breeds raised under hot climate in the gulf desert lack the gene of fecundity. Moreover, they rarely produce twins or multiple due to the harsh condition of high temperature and lack of feedstuffs. Nutrition during ewe pregnancy is of critical importance. Administration of L-arginine during various stages of gestation to enhance maternal health and fetal birth weight has drawn the attention of several researchers [5].

Recently, several attempts have been made to induce twinning in the single-bearing ewes. Of these, Najafi *et al*. [6] reported a higher twinning rate by 23% in the controlled internal drug release (CIDR) pregnant mare serum gonadotropin-treated ewes than control ewes given saline. Several protocols are implemented to induce twinning in sheep using various preparations, sources and dosages of follicle-stimulating hormone (FSH). Using a recombinant FSH for fertility improvement in *Awassi* ewes was primarily mentioned by Yavuzer *et al*. [7], who found about 50% increase in twinning rate compared with saline given ewes. In Boer goats, porcine FSH dose was found to be dependent on body weight (BW) resulting in the highest embryo outcome from 5 mg FSH per kg BW[8]. The hypothesis focuses on the possibility of the interaction of a recombinant human FSH (rh-FSH)-/porcine FSH with ovine granulosa to induce twinning and the consequences on the maternal blood parameters in indigenous ewes.

Recently, Abecia and Palacios [9] found that ewes giving birth to twin lambs produced more milk than ewes giving birth to single lambs. Extra source of energy must be provided during the last trimester of the gestation, especially for the multiple-bearing ewes[10].

Therefore, the main goal of the present study was to evaluate the effectiveness of a twinning dose of rh-FSH as compared to the porcine FSH either given as a single dose or six doses on the prolificacy traits and maternal blood constituents of *Noemi* ewes.

## Materials and Methods

### Ethical approval

The ethical use of the animals was applied and approved according to the committee guideline of the animal use, care, and handling of Qassim University, Saudi Arabia.

### Animals’ management

Sixty adult healthy *Noemi* ewes were housed in the shaded barn during January and October 2017 (average temperature between 19 and 44°C). Ewes were offered a commercial pelleted concentrate of soybean, corn, and barley containing 15% crude protein and 65% TDN (Saudi Silos Co.). At the first 50days of pregnancy, each ewe was given 350g/ewe/day and supplemented with 75mg/kg BW of L-arginine. At the mid-term of pregnancy (i.e.,second 50days), 400g of the pellets were offered per head per day without L-arginine and at the last trimester of pregnancy, each ewe was given 500g pellets plus 50ml of date molasses (20%) as a source of simple carbohydrates. In addition, alfalfa, clean water, and balanced mineral licks were offered as free choices. The study was conducted at the Agriculture Experimental Station, College of Agriculture and Veterinary Medicine, Qassim University, KSA.

### Experimental design

Ewes were inserted with controlled intravaginal device release (CIDR^®^ device; inter-Ag, New Zealand) for 10days. At day 8 of CIDR insertion, ewes were randomly allotted into six groups (n=10/group); C1 ewes served as control given one dose (i.m.) of saline, C6 ewes were given 6 doses of saline, H1 ewes were given 180IU rh-FSH (rh-FSH; GONAL-F, Merck Sharp and Dohme) as one dose at day 8 (a.m.), H6 ewes were given 40, 40, 30, 30, and 20, 20IU at days 8, 9, and 10 (a.m. and p.m.), respectively, P1 ewes were given one dose of 133.33mg *p*FSH [(*p*FSH; Folltropin-V Emballage double, Vetoquinol, Canada); equivalent to 180IU rh-FSH] at day 8 (a.m.), and P6 ewes were given as 26.66, 26.66, 22.85, 22.85, and 17.15, 17.15mg at days 8, 9, and 10 (a.m. and p.m.), respectively. All ewes were given 180IU *h*CG (Pregnyl 5000IU, Merck Sharp and Dohme) at the fifth dose (day 10 a.m.). The dose of *h*CG was given to the H1, H6, P1, and P6 ewes only; however, C1 and C6 ewes were concomitantly administered with saline. CIDR was removed from the ewes on day 10 (a.m.). Fertile ram was introduced with the ewes after CIDR withdrawal. Percent of females exhibited estrus was determined within a group. Furthermore, the time interval from CIDR withdrawal until the onset of estrus was assessed. Pregnancy was determined 30days after natural mating using a transrectal 5 MHz probe ultrasonography (Aloka SSD 500, Tokyo, Japan).

### Blood sampling and serum harvesting

Blood samples in anticoagulant tubes for blood chemistry were collected from three ewes within a group just before CIDR insertion, at CIDR removal, and thereafter once daily until and during estrus and continued thereafter once a week until lambing. In case of the non-appearance of estrus signs in a ewe designed for blood collection within a group, it was replaced by another ewe within the same group. Plasma was harvested after centrifugation at 1300× g for 15min in a cold condition (5°C), stored frozen (−20°C) until analyzed for blood biochemical attributes.

### Ultrasound measurements

Ultrasound (Aloka SSD 500, Japan) diagnoses of ovarian structures were performed to all ewes at CIDR insertion, removal, and then daily until the estrus exhibition. Follicle numbers and sizes in both ovaries were determined. In addition, 30days after natural mating, all ewes were ultrasonically examined for pregnancy diagnosis.

### Blood biochemical attributes determination

#### Total protein determination

Serum total protein was determined by the Biuret method (Human, Liquicolor, Germany), according to Weichselbaum [11].

#### Albumin and globulins determination

Serum albumin was quantified by the bromocresol green (BCG) method (BCG, Human, Liquicolor, Germany), according to Rodkey [12]. Total globulins were estimated mathematically by subtracting the value of albumin from the value of total proteins.

#### Glucose determination

Serum glucose was determined by the glucose oxidase-peroxidase (PAP) method (Human, Liquicolor, Germany), according to Barham and Trinder [13].

#### Low-density lipoprotein (LDL) cholesterol determination

Serum LDL concentration was determined by the enzymatic colorimetric test (Human, Liquicolor, Germany), according to Okada *et al*. [14].

#### High-density lipoprotein (HDL) cholesterol determination

Serum HDL was determined by the enzymatic colorimetric method (Human, Liquicolor, Germany), according to Gordon *et al*. [15].

#### Cholesterol determination

Serum cholesterol determination was achieved by the cholesterol oxidase-PAP method (Human, Liquicolor, Germany), according to Carr *et al*. [16].

#### Triglycerides (TGs) determination

Serum TGs were determined by the glycerol-3-phosphate oxidase-PAP method (Human, Liquicolor, Germany), according to Carr *et al*. [16].

#### Aspartate aminotransferase (AST) determination

Serum activity of AST was determined (Human, Liquicolor, Germany), according to Schumann *et al*.[17].

#### Alanine aminotransferase (ALT) determination

Serum activity of ALT was determined (Human, Liquicolor, Germany), according to Schumann *et al*. [18].

### Statistical analysis

Data for estrous and time from CIDR removal to onset of estrus and neonatal traits were analyzed by least square analysis of variance [19]. Data of blood biochemical attributes were analyzed by least square analysis of variance for repeated measures using SAS package. The model of statistical analysis used in analysis was as follows:


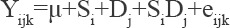


Y_ijk_=The observation taken on the k^th^ individual;

μ=Overall mean;

S_i_=Effect of source of FSH (human vs. porcine);

D_j_=Effect of number of FSH injections (one vs. six injections);

S_i_D_j_=Interaction between source and number of FSH injections;

e_iJk_=Random error assumed to be independent normally distributed with mean=0 and variance=σ^2^.

Differences between treatment means were achieved by the Duncan’s Multiple Range Test [20]. Significant differences were considered at p<0.05.

## Results

### Estrous exhibition

The percentage of estrous display was different among treatments. The highest estrous exhibition was found in H1(10/10; 100%) and P1(10/10; 100%); however, the control animals displayed the least percent of estrous (12/20; 60%). On the other hand, P6 and H6 ewes exhibited 80% (8/10) and 90% (9/10) estrous signs, respectively. Injection of either human or porcine FSH increased percentage of ewes showing estrus by 30-35% over the control. Combining FSH in one dose induced sharp display of estrous signs (100% in P1 and H1), while splitting FSH dose into six injections slightly reduced this percentage (i.e.,80% and 90% in P6 and H6, respectively).

### Time interval from CIDR removal to onset of estrus

Time from CIDR removal to onset of estrus was variable (p<0.05) among treatments. The longest interval was found in C ewes (63h). However, P1 ewes showed an intermediate (46.7h) interval. On the other hand, H1, H6, and P6 ewes showed shorter intervals (26.6, 25.3, and 28.2h, respectively). In general, human FSH was most effective than porcine FSH in reducing number of hours from CIDR removal until onset of estrus with mean intervals 26.3 and 38.3h in human and porcine FSH-treated ewes, respectively.

### Follicular dynamics

A number of follicles in ovaries of treated ewes was higher (p<0.05) than in control. The folliculogenesis response to *human* FSH was higher than for *porcine* FSH. Anumber of ovarian follicles increased by time from CIDR insertion until its removal and estrous displays. Furthermore, the largest follicle diameter at onset of estrus was found in the ewes given six doses of human FSH (H6; 6.32mm) compared with control (4.77mm). There were no significant differences in follicular diameters due to other treatments.

### Pregnancy rate and neonatal traits

The pregnancy rate increased in FSH-treated ewes (mean 55%) compared with control (mean 35%). Percent of ewes giving birth to twins was 0, 75, 80, 100, and 50% in C, H1, H6, P1, and P6, respectively. Moreover, survival rate at birth was 100, 18.2, 40, 33.3, and 25% in C, H1, H6, P1, and P6, respectively. Mean lamb birth weight was 5.2, 2.7, 3.8, 3.4, and 5.1kg in C, H1, H6, P1, and P6 ewes, respectively. The 90-day weaning weight was 28.1, 19.5, 28.3, 18.0, and 28.2kg in C, H1, H6, P1, and P6, respectively. The average daily gain between birth and weaning was 254, 180, 275, 165, and 255g/day in C, H1, H6, P1, and P6, respectively.

### Blood biochemical attributes

**Figure-1:**
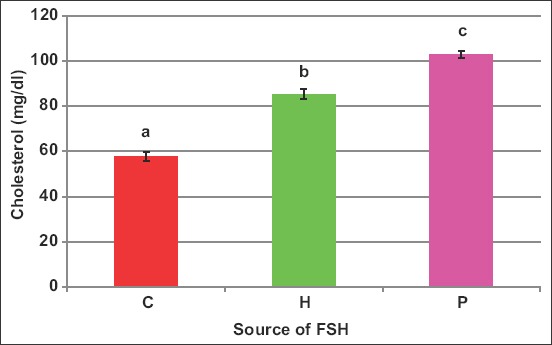
Effect of the source of follicle-stimulating hormone (*human* vs*. porcine*) on blood cholesterol of *Noemi* ewes induced for twinning ([p<0.05, H=Human FSH, P=Porcine FSH]).

**Figure-2:**
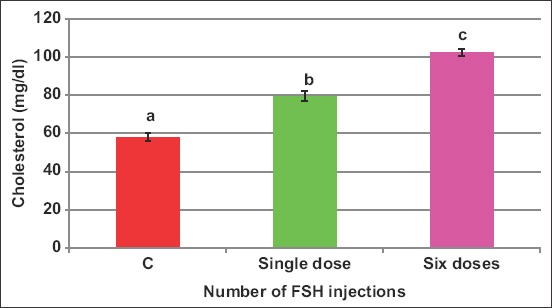
Effect of the number of doses of follicle-stimulating hormone on blood cholesterol of *Noemi* ewes induced for twinning (p<0.05).

There exists an interaction (p<0.05) between FSH source and number of injections on blood cholesterol. Cholesterol concentration increased by FSH administration of both sources with values of 57.9, 85.5, and 103.2mg/dL in C, H, and P, respectively (Figure-1). Moreover, the protocol of six injections increased (p<0.05) cholesterol (102.3mg/dL) than the one-dose protocol (79.4mg/dL) and both raised cholesterol than in control (57.9mg/dL) ewes (Figure-2).

**Figure-3:**
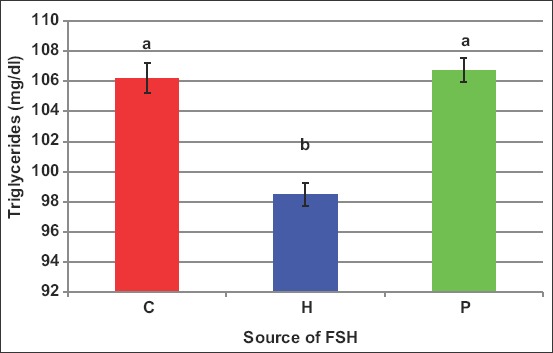
Effect of the source of follicle-stimulating hormone on blood triglycerides of *Noemi* ewes induced for twinning ([p<0.05, H=Human FSH, P=Porcine FSH]).

**Figure-4:**
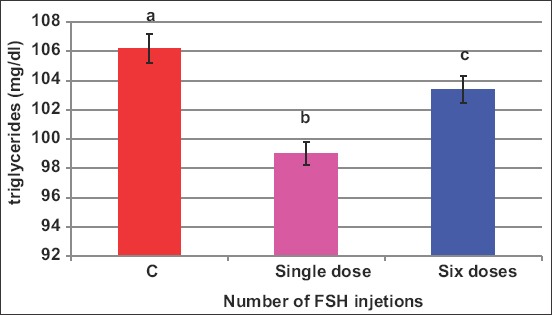
Effect of the number of follicle-stimulating hormone injections on blood triglycerides of *Noemi* ewes induced for twinning (p<0.05).

Human FSH reduced (p<0.05) peripheral TG (Figure-3) from 106.2mg/dL in control to 98.5mg/dL; however, porcine FSH did not change this parameter (106.7mg/dL). This reduction was observed in case of one or six injections (Figure-4).

**Figure-5:**
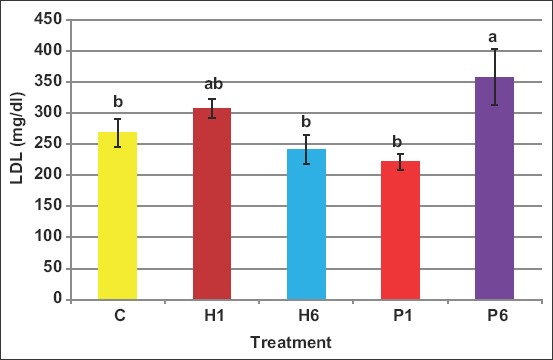
Effect of the source of follicle-stimulating hormone and series of injections on blood low-density lipoprotein of *Noemi* ewes induced for twinning ([p<0.05, C=Control, H1=One-dose human FSH, H6=Six doses, P1=One-dose porcine FSH, P6=Six doses FSH]).

Blood LDL levels were not affected due to treatments, except it increased in case of P6 (Figure-5). Values of LDL were 269.1, 307.5, 242.2, 221.8, and 358.8mg/dL in C, H1, H6, P1, and P6, respectively.

**Figure-6:**
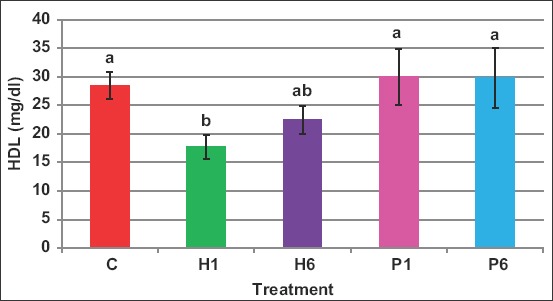
Effect of the source of follicle-stimulating hormone and series of injections on blood high-density lipoprotein of *Noemi* ewes induced for twinning ([p<0.05, C=Control, H1=One-dose human FSH, H6=Six doses, P1=One-dose porcine FSH, P6=Six doses FSH]).

HDL concentrations were not different due to treatments, except in case of H1, which reduced the HDL level from 28.4mg/dL in control to 17.7mg/dL, representing 37.7% reduction (Figure-6).

**Figure-7:**
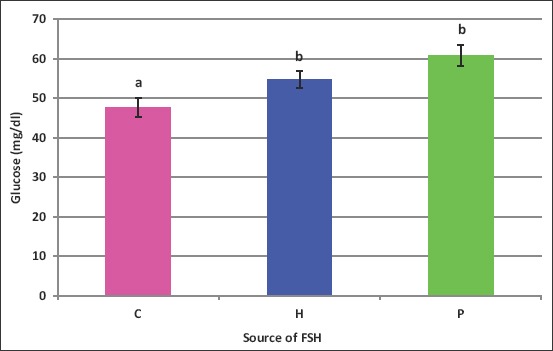
Effect of the source of follicle-stimulating hormone (*human* vs. *porcine*) on blood glucose of *Noemi* ewes induced for twinning ([p<0.05, H=Human FSH, P=Porcine FSH]).

**Figure-8:**
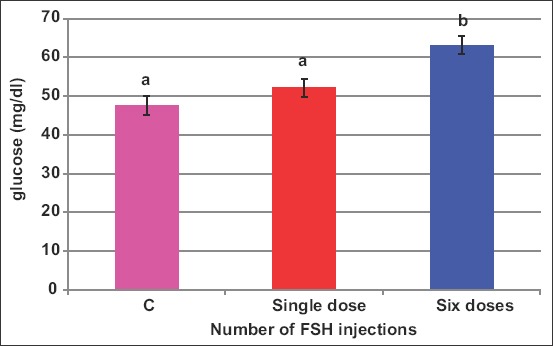
Effect of the number of follicle-stimulating hormone injections on blood glucose of *Noemi* ewes induced for twinning (p<0.05).

Both FSH sources increased (p<0.05) blood glucose (Figure-7) when given in a split six doses; however, when either source of FSH was given in one dose, there were no changes in blood glucose (Figure-8). The values of glucose were 47.6, 45.4, 65.2, 67.2, and 56.2mg/dL in C, H1, H6, P1, and P6 ewes, respectively.

**Figure-9:**
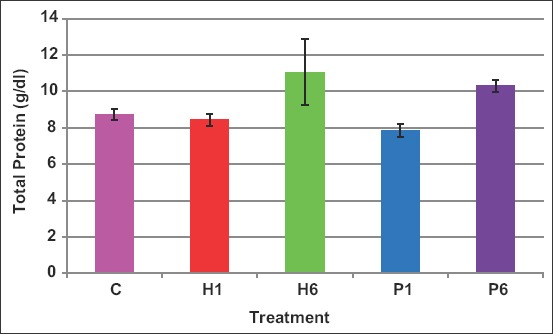
Effect of the source of follicle-stimulating hormone and series of injections on blood protein of *Noemi* ewes induced for twinning ([p>0.05, C=Control, H1=One-dose human FSH, H6=Six doses, P1=One-dose porcine FSH, P6=Six doses FSH]).

Total protein concentrations in blood increased (p<0.05) compared with control ewes only when FSH of either source was given in six doses (Figure-9). The values of total protein were 8.7, 8.5, 11.0, 7.9, and 10.3mg/dL in C, H1, H6, P1, and P6 ewes, respectively.

**Figure-10:**
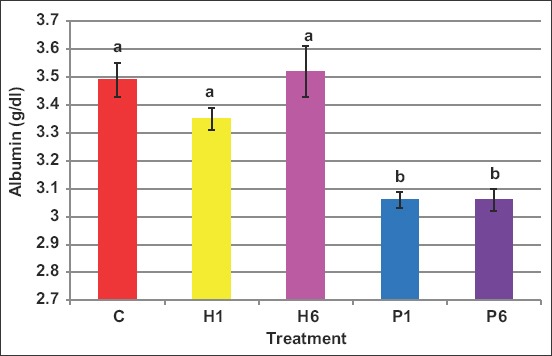
Effect of the source of follicle-stimulating hormone and series of injections on blood albumin of *Noemi* ewes induced for twinning ([p<0.05, C=Control, H1=One-dose human FSH, H6=Six doses, P1=One-dose porcine FSH, P6=Six doses FSH)].

Blood albumin levels were not affected by human FSH, whereas porcine FSH reduced (p<0.05) albumin (Figure-10) with values being 3.5, 3.4, 3.6, 3.1, and 3.1mg/d Lin C, H1, H6, P1, and P6, respectively.

**Figure-11:**
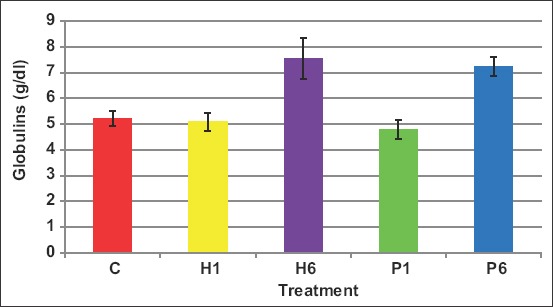
Effect of the source of follicle-stimulating hormone and series of injections on blood globulins of *Noemi* ewes induced for twinning ([p>0.05, C=Control, H1=One-dose human FSH, H6=Six doses, P1=One-dose porcine FSH, P6=Six doses FSH)].

Globulins levels in blood were affected by the protocol of injection as it increased (p<0.05) when FSH of either source was administered as six doses (Figure-11). The levels of blood globulins were 5.2, 5.1, 7.5, 4.8, and 7.2mg/dL in C, H1, H6, P1, and P6 ewes, respectively.

**Figure-12:**
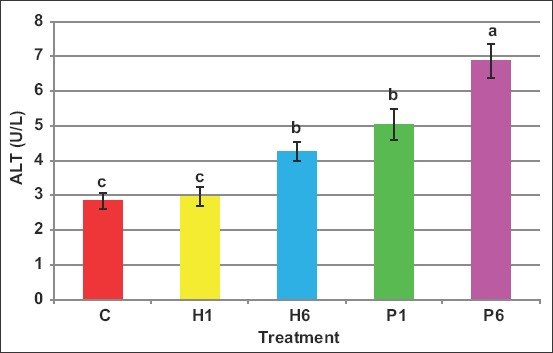
Effect of the source of FSH and series of injections on ALT in blood of *Noemi* ewes induced for twinning ([p<0.05, C=Control, H1=One-dose human FSH, H6=Six doses, P1=One-dose porcine FSH, P6=Six doses FSH]).

The activity of ALT increased by FSH injection and this increase was evident when FSH of either source was given in six doses (Figure-12). The activity values in C, H1, H6, P1, and P6 were 2.8, 2.9, 4.3, 5.0, and 6.9IU/L, respectively.

**Figure-13:**
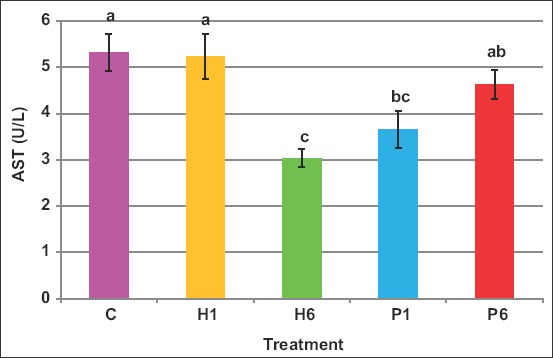
Effect of the source of FSH and series of injections on AST in blood of *Noemi* ewes induced for twinning ([p<0.05, C=Control, H1=One-dose human FSH, H6=Six doses, P1=One-dose porcine FSH, P6=Six doses FSH]).

On the other hand, FSH of either source reduced the AST activity (Figure-13). The respective activity values in C, H1, H6, P1, and P6 were 5.3, 5.2, 3.0, 3.7, and 4.6IU/L, respectively.

## Discussion

Lack of genes responsible for the high prolificacy in indigenous sheep breeds in the Gulf region imposed burdens on the animal scientists to find out alternatives for maximizing the productivity of the animal unit [1]. *Noemi* sheep is an indigenous breed raised in the Gulf area characterized by low prolificacy (litter size of 1.2). The induction of twinning is another way to maximize the profitability of raising this precious breed. Due to the fact that FSH binds to the ovarian follicular granulosa cells, this leads to a cascade of kinetic reactions [21]. The purity of the rh-FSH due to its production for human uses has shown better responses in ewes given a proper dose to produce live neonates. Evidently, the specificity of the human FSH molecules has promptly raised the ovarian follicular estrogens, leading to the acceleration of estrous exhibition as seen in the H1 and H6 groups. In the current study, the administration of FSH of either source (*porcine* and *human*) increased estrogens secretion (100.15±20.9, 219.29±42.8, and 175.95±56.5pg/mL in C, H, and P, respectively). The rapid proliferation of the pre-ovulatory dominant follicles due to the excess exogenous FSH leads to the secretion of estrogens showing estrous signs [22]. Combining FSH in one dose induced sharp display of estrous signs (100% in P1 and P6), while splitting the FSH dose into six injections slightly reduced this percentage (i.e.,80-90%). The gradual elevation of FSH in blood of ewes given six injections might explain the shorter CIDR removal-estrus intervals, which were seen in H1, H6 and P6 treatments. This finding confirms the purity of the rh-FSH molecules surpassing that of *porcine* FSH, resulting in similar short intervals to that found in H6 and P6 treatments. Pregnancy rates relatively increased in H1, H6, and P1 as compared with control ewes. On the contrary, P6 ewes revealed less pregnancy due to that most of the ewes within this group were not responsive to the treatment at the beginning, and that delay caused the postponement of the pregnancy attaining the hot summer. Hudson *et al*. [23] indicated that exogenous FSH increased ovulation rates and pregnancy in Booroola ewes after hypothalamic-pituitary disconnection or after treatment with a gonadotropin-releasing hormone (GnRH) agonist. In a study on dairy cows, Ali and Zeitoun [24] reported that injection of 525IU rh-FSH increased pregnancy rate from 60% in control to 100%, however, the injection of overdose (1800IU) suppressed the ovulation and conception. In a study on *Awassi* sheep, the researchers found that the administration of a recombinant FSH, 24h before the second PGF2α injection led to an increase in twin pregnancy rate [7]. In addition, others found that the administration of 10IU FSH at the end of progestagen treatment (i.e., at sponge removal) resulted in higher lambing rate and higher mean number of lambs born per a ewe [25]. Using 10-day CIDR insertion for estrous synchronization was long enough to prepare the ewe’s reproductive system for the FSH actions. This concept was recently confirmed by Martinez-Ros *et al*. [26].

The supplementation of ewes with a source of protein at their early pregnancy and with a source of simple sugars at late pregnancy not only maintained the maternal health well-being but it also increased milk production (1.15±0.08vs. 1.35±0.12kg milk/day in control vs. treated ewes) for better twin birth growth. This approach was effective in maintaining the sheep maternal health well-being during gestation and resulted in heavier lambs at birth [27]. In case of the single dose of either hormonal source in the current study, this is most likely caused congestions of the fetuses due to the simultaneous ovulations. This congestion not only caused less opportunity for each embryo to find a space for its growth and development but it also resulted in higher stillborn lambs, mummified embryos, and even produced lower birth weight lambs. Recently, Beede *et al*. [28] recognized various factors, including heat stress within the multiple-bearing uteri in sheep resulting in the incidence of intrauterine growth restriction. As twin birth weight increased, there found a decrease in the mortalities between birth and weaning [29]. Simultaneous ovulations could result in crowding within uterus and lack of enough nutrients supply for each embryo. Congestion of embryos within a limited uterine space might have a profound effect on placental and fetal development, possibly leading to compromised pregnancies associated with poor placental development [30]. There also appears to found immune factors at an early gestation that might regulate the future embryonic development within uterus [31]. The differences in the responses due to the source of FSH would be explained from the point of the purity and specificity of the FSH molecules derived by genetic engineering. Clinical experience has shown the higher effectiveness of rh-FSH at stimulating ovarian follicular growth than urinary and pituitary FSH [32]. Accordingly, the recent progress of the biotechnology to produce pure specific molecules of FSH enhanced the results of reproductive performance in human as well as aquatic and mammalian species [33].

In human, researchers found that women exposed to a recombinant FSH for ovarian stimulation had displayed increases of cholesterol, LDL, TG, and apolipoprotein [34]. Concurrently, Chu *et al*. [35] in their study on the pre-and post-menopausal women found that increased normal levels of FSH >7IU/L resulted in elevations of some lipid fractions (i.e., cholesterol and LDL, but not TG or HDL) compared with their levels when FSH levels were <7IU/L. This finding coincides with our results that show increases in total cholesterol due to external FSH (*porcine* or *human*) administration. Neither type of FSH nor number of injections affected LDL concentrations in the present study. Contrariwise, both *human* and *porcine* FSH reduced triglyceride levels in peripheral blood. In a study on human, the authors used clomiphene citrate (i.e., a selective estrogen receptor modulator) to lessen the internal FSH secretion; they did not find alterations in the levels of cholesterol, LDL, TG, or HDL [36]. The potency of FSH to increase blood cholesterol was raised by the existence of LH molecule. Moreover, segmenting the dose of FSH into six injections might gave an opportunity for the liver (i.e., time wise) to build up further cholesterol as one dose of the *human* FSH has not shown this effect. The impact of *porcine* FSH on the elevation of peripheral glucose concentrations was about twice that found with *human* FSH. Park and Chun correlated the LH/FSH ratio with metabolic parameters in polycystic ovarian syndrome (PCOS) women and they concluded that most parameters correlated with insulin resistance [37]. It has also been suggested recently that high LH/FSH impaired insulin sensitivity resulting in elevations of blood glucose; this was confirmed in PCOS obese women [38]. In an earlier study, there found a significant increase in blood glucose when women were injected with GnRH[39]. Mild changes were found in total protein, albumin, and globulins. The increases of total protein and globulins throughout gestation were attributed to the anabolic effects of the female sex steroid hormones (i.e., estrogens and progesterone) on the hepatic anabolic ability [40]. It is of interest to point out that protein and its fractions (i.e., albumin and globulins) are indispensable requirements in fertilization and embryo development *in vitro* [41]. The protocol of splitting the FSH dose into six injections has shown better production of globulins on account of albumin. Looking at the AST/ALT ratio in the present study, the treatments resulted in similar hepatic enzyme activities, being indicative of liver cellular integrity. McGovern *et al*. [42] have proposed that an AST/ALT ratio of ≤0.4 during recovery from severe acute hepatotoxicity indicates a good prognosis for recovery. On the other hand, some clinicians considered that the AST/ALT ratio of 2:1 is an indicative of hepatic injury[43]. The clinical importance for the measurements of the liver enzymes is of critical value to evaluate the effect of the treatments on general health. The previous reports indicated that repeated ovarian stimulation might deteriorate the hepatic functions, leading to liver failure [44]. There have been variations in ALT and AST activities due to animal physiological status. Stojevic *et al*. [45] stated that as dairy cows progressed in their lactation days, there existed sharp declines in AST and ALT activities. The temporal changes of liver enzymes depending on the physiological stage have been shown recently in dairy cows[46]. This author found a similar trend of the activities of ALT increase and AST decrease in the blood and milk of dairy cows.

The cost-effectiveness of this study to the sheep raiser obviously revealed that the highest numbers of weaned lambs were obtained in H6 (i.e., six descending doses of human FSH). The market price and the number of live weight kilograms of lambs obtained out of each treatment were taken in mind. At weaning, the treatments resulted in 3, 4, 2, 3, 2, and 7 live weaned lambs/treatment, with respective weaning weights of 28.3, 27.2, 18, 28.2, 19.5, and 28.3kg/lamb in C1, C6, P1, P6, H1, and H6. Altogether, these resulted in 84.9, 108.8, 36.0, 84.6, 39, and 198.1kg lambs/treatment. Therefore, the results indicate that the best treatment to be adopted by the sheep raiser is H6 resulting in the best profit. Feasibility study revealed that, considering the cost of the CIDR and hormones and estimating the return of the offspring price, there found profit amounts of 38, 54, −10, 6, −10, and 70 USD/treated ewe in C1, C6, P1, P6, H1, and H6 respectively.

## Conclusion

The induction of twinning in single-bearing *Noemi* ewes is of economic feasibility. To optimize a protocol for the sheep owners, there must use a 10days CIDR insert followed by six descending doses of a total of 180IU FSH accompanied by an equal dose of *h*CG. Moreover, using the rh-FSH was found to be more effective than porcine FSH in this respect. Even though, the treatments showed a higher percent of mortalities, this protocol not only resulted in good healthy neonates attaining the weaning age similar to the control single beard lambs, it also maintained the maternal health well-being of *Noemi* ewes. The best survival for mothers and neonates was attained by providing the ewes with a source of protein in the first trimester and a source of simple carbohydrates at the last trimester.

## Authors’ Contributions

MMZ created the study concept and design, helped in the laboratory measurements and wrote the final draft of the manuscript. AOE carried out the fieldwork and helped in the laboratory work. MAA carried out the field tasks. MASE carried out the data analyses. All authors read and approved the final manuscript.
